# Seneca Valley virus 3C^pro^ antagonizes host innate immune responses and programmed cell death

**DOI:** 10.3389/fmicb.2023.1235620

**Published:** 2023-10-06

**Authors:** Xin-yu Zhang, Yu-ying Li, Hai-xin Huang, Chen-chen Zhao, Xiao-xiao Lei, Bao-peng Zhao, Jing-yi Lu, Tian Lan, Wen-chao Sun

**Affiliations:** ^1^Institute of Virology, Wenzhou University, Wenzhou, China; ^2^College of Veterinary Medicine, Northwest A&F University, Xianyang, China

**Keywords:** 3C protease, virus replication, interferon pathway, host RNA metabolism, programmed cell death

## Abstract

Seneca Valley virus (SVV), a member of the *Picornaviridae* family, may cause serious water blister diseases in pregnant sows and acute death in newborn piglets, which have resulted in economic losses in pig production. The 3C protease is a vital enzyme for SVV maturation and is capable of regulating protein cleavage and RNA replication of the virus. Additionally, this protease can impede the host’s innate immune response by targeting the interferon pathway’s principal factor and enhance virus replication by modulating the host’s RNA metabolism while simultaneously triggering programmed cell death. This article reviews recent studies on SVV 3C functions, which include viral replication promotion, cell apoptosis modulation and host immune response evasion, and provides a theoretical basis for research on preventing and controlling SVV infection.

## 1. Introduction

In 2002, laboratory personnel at Gaithersburg, Maryland, United States, serendipitously discovered the Seneca Valley virus (SVV) when culturing a vector derived from adenovirus-5 (Ad5) in PER.C6 cells (Hales et al., 2008). Research has revealed that SVV exerts an oncolytic effect that holds promising prospects for the treatment of neuroendocrine tumours in humans (Reddy et al., 2007). SVV infection can result in vesicular disease in sows and sudden neonatal piglet mortality. The initial stages of SVV may be characterized by anorexia, lethargy, and fever, and these signs are followed by vesicular lesions presenting on the skin and the mucous membranes of the hoof, mouth, and nose, which may cause mouth and nose ulceration and even hoof horn loss and thus lead to lameness in severe cases. The symptoms of SVV are similar to those of foot-and-mouth disease virus (FMDV) infection and the two viral infections can only be distinguished by laboratory diagnosis (Reddy et al., 2007; Joshi et al., 2016; Maggioli et al., 2018; Wu et al., 2022). SVV has been spreading in pig herds in the United States since 1988, but water blister disease caused by SVV was not found until 2007 (Knowles et al., 2006; Pasma et al., 2008). Sporadic cases of SVV infection have been reported for more than 10 years since the discovery of this virus; however, a sudden outbreak was reported in Brazil at the end of 2014 and the beginning of 2015 (Vannucci et al., 2015; Leme et al., 2016) and a rapid and large-scale spread of SVV to countries such as the United States (Hause et al., 2016), Canada (Xu et al., 2017), Colombia (Sun et al., 2017), China (Wu et al., 2017), Thailand (Saeng-Chuto et al., 2018), and Vietnam (Arzt et al., 2019) then occurred. Notably, SVV frequently causes water blister disease outbreaks in the pig herds of the world’s leading pig-producing countries.

SVV, which belongs to the *Picornaviridae* family and the *Senecavirus* genus, is an unenveloped single-stranded positive-sense RNA virus. The viral genome consists of an ORF, a 5′ untranslated region (5′ UTR), and a 3′ untranslated region (3′ UTR). The viral genome initially encodes a polyprotein that is then cleaved into structural and nonstructural proteins during cotranslational and posttranslational processes. Structural proteins comprise VP1, VP2, VP3, and VP4, whereas nonstructural proteins comprise 2A, 2B, 2C, 3A, 3B, 3C, and 3D as well as the leader protein L (Hales et al., 2008; Meng et al., 2022). The nonstructural protein 3C is a cysteine protease encoded by the virus that has a conserved catalytic triad of His, Asp., and Cys (Meng et al., 2022). This protease has been shown to be involved in various pathological processes of SVV, and SVV 3C^pro^ employs complex strategies to modulate host antiviral immunity, which is essential for effective virus replication.

## 2. Structure of the 3C protease and virus amplification

Similar to the 3C^pro^ of other *Picornaviridae* viruses, SVV 3C^pro^ adopts the typical trypsin fold, but it lacks the characteristic KFRDI motif. H48 is one of the catalytic residues, and G158 and G161 are typical cysteine protease motifs with the sequence G-X-C/S-G (Ng et al., 2021; Meng et al., 2022). Furthermore, the catalytic cysteine residue typically participates in substrate recognition with the downstream GΦH motif (SVV^GLH^ 3C^176–178^), which consists of 10–20 amino acids (Hales et al., 2008).

The SVV genome has an open reading frame encoding a polyprotein that consists of a leader protein (L), a structural protein region (P1) and nonstructural protein regions (P2 and P3) (Hales et al., 2008; Meng et al., 2022). The virus-encoded 3C^pro^ cleaves the polyprotein into its active structural and nonstructural proteins, and RNA replication is a crucial stage in virus maturation (Pathak et al., 2007). The structural protein region P1 undergoes cleavage, which produces VP0, VP3, and VP1. The precursor VP0 undergoes subsequent cleavage to yield VP4 and VP2, and the P2-P3 region undergoes cleavage, leading to the formation of 7 nonstructural proteins: 2A, 2B, 2C, 3A, 3B, 3C, and 3D. Virus-encoded 3C^pro^ cleaves specific sites, such as VP2-VP3, VP3-VP1, 2B-2C, 2C-3A, 3A-3B, 3B-3C, and 3C-3D. However, the cleavage mechanisms of L-VP4, VP1-2A, and 2A-2B differ among distinct *Picornaviridae* viruses. SVV 3C^pro^ cleaves both L-VP4 and VP1-2A, whereas 2A-2B cleavage relies on 2A^pro^. Unfortunately, the cleavage mechanism of VP4-VP2 remains elusive (Hales et al., 2008; Meng et al., 2022).

Picornaviruses contain a VPg protein that is covalently linked to the 5′ end of their genome. During replication of the genome of picornaviruses, VPg undergoes uridylylation through oriI to form VPg-pUpU, which is believed to enhance the replication of viral RNA. Either the precursor protein 3CD or its derivative 3C triggers this reaction. Both 3CD and 3C contain RNA binding and proteinase activities. Research has shown that the 3C domain exhibits specificity for OriI, whereas the 3D domain enhances its overall affinity. OriI binds to 3C(D) and recruits a polymerase to this site (Pathak et al., 2007). 3C^pro^ has a crucial function in the *Picornaviridae* virus maturation process.

## 3. SVV 3C functions in host antiviral immunity

### 3.1. Role of 3C proteases in regulating mRNA

The DEAD-box protein family is a conserved family of RNA helicases containing a DEAD-box domain, named after the conserved Asp-Glu-Ala-Asp (DEAD) sequence (Pathak et al., 2007). DDX21 mediates innate immunity and regulates the viral replication of viruses such as foot-and-mouth disease virus (FMDV) (Abdullah et al., 2021), dengue virus (DENV) (Dong et al., 2016) and Borna disease virus (BDV) (Watanabe et al., 2009). Furthermore, DDX21 has been shown to inhibit SVV replication, but its effects can be weakened by SVV 3C^pro^, which induces caspase-dependent degradation of DDX21 and suppresses host antiviral immunity, limiting the cell antiviral response (Zhao et al., 2022). DHX30, a DEAD family member, contributes to the biosynthesis of mitochondrial ribosomes. Zinc-finger antiviral protein (ZAP) recruits DHX30 to unfold and degrade viral RNA (Ye et al., 2010). Although DHX30 inhibits SVV replication through its helicase activity, SVV 3C^pro^ can mediate the cleavage of DHX30 helicase at the Q220 site, leading to loss of its ability to inhibit SVV replication (Wen et al., 2022).

hnRNPs are a widely functional family of RNA-binding proteins mainly located in the cell nucleus (Jean-Philippe et al., 2013), hnRNP A1 can bind to viral proteins and modulate the replication of viruses such as the nucleocapsid proteins of porcine epidemic diarrhoea virus (PEDV) (Li et al., 2018). In addition, hnRNP A1 can interact directly with viral RNA sequences, including the coding region of the human papillomavirus 16 (HPV16) E7 sequence (Zheng et al., 2020). Research has shown that the protease activity of SVV 3C^pro^ mediates the degradation and translocation of hnRNP A1, which enhances the replication of SVV (Song et al., 2021). Similarly, hnRNP K participates in virus replication through interaction with the 5′ UTR of enterovirus 71 (EV71) (Lin et al., 2008) and FMDV (Liu et al., 2020). Knockdown of hnRNP K significantly inhibits SVV replication, whereas hnRNP K overexpression promotes virus proliferation. These studies suggest that intracellular hnRNP K contributes to SVV replication. SVV infection leads to the cleavage, degradation, and cytoplasmic redistribution of hnRNP K due to the activity of 3C^pro^. 3C^pro^ induces the degradation of hnRNP K through the caspase pathway and cleaves hnRNP K at Q364. The cleaved fragment hnRNP K (365–464) promotes virus replication, whereas full-length hnRNP K exerts a similar effect. In contrast, the noncleaved fragment hnRNP K (1–364) tends to inhibit virus replication (Song et al., 2022b).

Nucleolin (NCL) is involved in several cellular processes, such as RNA transcription, ribosome formation, nuclear-cytoplasmic transport, and posttranscriptional regulation of mRNA (Becherel et al., 2006; Durut and Sáez-Vásquez, 2015). Moreover, NCL is linked with viral proliferation and plays a crucial role in virus replication through nucleocytoplasmic redistribution (Greco et al., 2012; Han et al., 2021). An increase in the NCL expression levels and its cleavage are induced by SVV, which drives NCL redistribution outside the cell nucleus. The 3C^pro^ protein of SVV relocates NCL to the cytoplasm and cleaves it at Q545. Cleaved NCL facilitates virus replication, and the proteolytic activity of the 3C^pro^ protein regulates the cleavage and relocalization of NCL (Song et al., 2022c).

Cytoplasmic poly (A)-binding protein 1 (PABPC1) interacts with eukaryotic translation initiation factor 4G (eIF4G) and promotes the binding of the 60S ribosomal subunit to the 48S preinitiation complex in the final step of initiation, facilitating translation initiation (Tahiri-Alaoui et al., 2014; Smith et al., 2017). SVV replication is inhibited by PABPC1; however, SVV 3C^pro^ cleaves PABPC1 at Q437, which disrupts protein synthesis and interferes with host immunity, thereby promoting virus replication (Xue et al., 2020).

SVV 3C^pro^ inhibits RNA metabolism regulation, gene expression, and the immune response mediated by the aforementioned factors, thereby evading host defence mechanisms and providing a conducive environment for its replication.

### 3.2. Role of 3C protease in cell intrinsic innate immunity signalling

The innate immune response represents the crucial initial defence against pathogenic invasion in the host organism. RIG-I-like receptors (RLRs), including retinoic acid-inducible gene I (RIG-I) and melanoma differentiation-associated protein 5 (MDA5), detect viral RNA and trigger the activation of mitochondrial antiviral signalling protein (Yoneyama et al., 2005; Paludan and Bowie, 2013). Upon activation, MAVS undergoes dimerization on the mitochondrial membrane, where it subsequently associates with TNF receptor-associated factor 3 (TRAF3) (Yoneyama et al., 2005; Tang and Wang, 2009). TRAF3 then recruits TRAF-related NF-κB-activating kinase (TANK), and TANK transduces upstream signals to TANK-binding kinase 1 (TBK1). TBK1 induces the phosphorylation of interferon regulatory factor 3/7 (IRF-3/7) and nuclear transcription factor kappa B (NF-κB), resulting in their dimerization and nuclear translocation (Pomerantz and Baltimore, 1999; Taniguchi et al., 2001; Shu et al., 2013). Ubiquitination is a crucial regulatory element in this process. For RIG-I to bind to RNA and become active, TRIM25 must undergo K63 ubiquitination. TRAF3 is K63-ubiquitinated, leading to the stimulation of IRF-3 phosphorylation (Castanier et al., 2012; Zhu et al., 2020). TIR domain containing adaptor molecule 1 (TRIF) responds to dsRNA and LPS, resulting in the activation of NF-κB (Schwandner et al., 1999; Alexopoulou et al., 2001).

**Figure 1:**
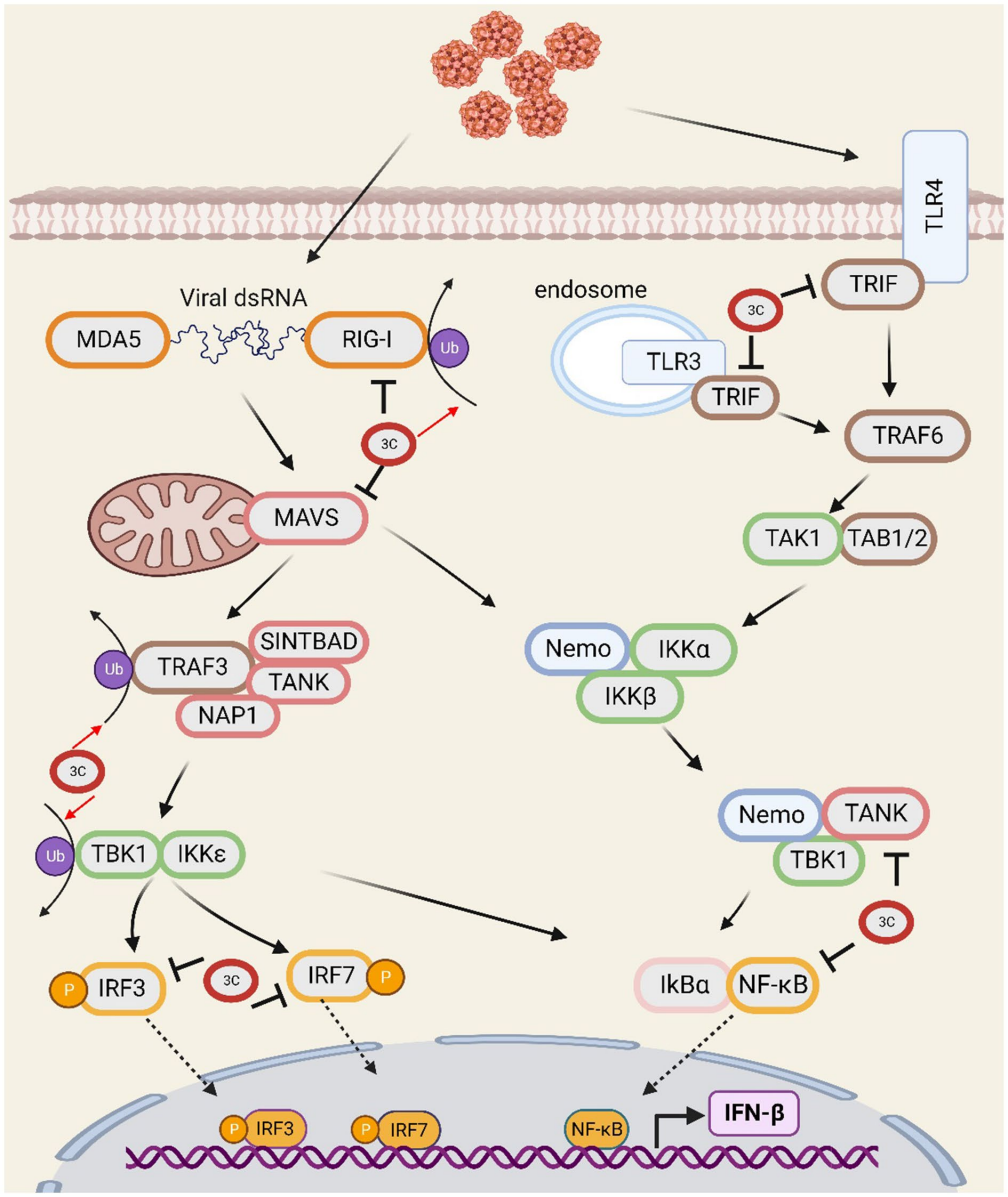
The SVV 3C protease is involved in the interferon signal transduction pathway and affects antiviral responses.

The 3C^pro^ of SVV extensively inhibits the type I interferon (IFN) pathway by impairing vital factors. SVV 3C^pro^ inhibits MAVS-mediated downstream signal transduction by degrading RIG-I, which is responsible for recognizing viral RNA. Second, by cleaving MAVS (Qian et al., 2017) at Q148, SVV 3C^pro^ interrupts its interaction with RIG-I (Wen et al., 2019), leading to reduced activation of downstream pathways. Recent studies have shown that SVV infection suppresses the interaction between MAVS and RIG-I by promoting lactate production through glycolysis, thereby facilitating virus replication (Li et al., 2023). SVV 3C^pro^ also possesses deubiquitinase activity, which inhibits the ubiquitination of RIG-I, TBK1, and TRAF3 (Xue et al., 2018b). In addition, SVV 3C^pro^ cleaves TRIF at Q159 (Qian et al., 2017). The N-and C-terminal domains of TRIF play different roles in the regulation of innate immunity and cell apoptosis. The N-terminal domain of TRIF is essential for the initiation of IFN promoter activity, whereas both the N-and C-terminal domains are involved in NF-κB activation. Despite the N-terminal domain separating from TRIF, the C-terminal domain still plays a role in initiating interferon signals, activating NF-κB, and inducing cell apoptosis. SVV 3C^pro^ interacts with IRF3 and IRF7, which inhibits their phosphorylation and leads to their degradation, ultimately affecting the regulation of IFN and other inflammatory genes (Xue et al., 2018a). In addition, SVV 3C^pro^ cleaves TANK at the E272 and Q291 sites (Qian et al., 2017), affecting its regulation of TBK1/IKKε and IRF3/7, and resulting in the inhibition of IFN production. Moreover, SVV 3C^pro^ mediates the cleavage of NF-κB by caspase 3 (Fernandes et al., 2019), suppressing its regulation of cytokine expression. SVV 3C^pro^ utilizes multiple mechanisms to deregulate crucial factors in the IFN pathway and thus widely inhibits the production and transfer of IFN, which enables it to evade the host’s immune response (Figure 1).

### 3.3. Role of 3C protease in stress granules

Stress granules (SGs) are cytoplasmic structures composed of RNA and RNA-binding proteins that form in response to various stressors, including hypoxia, environmental toxins, and viral infections (Wen et al., 2020). The formation of SGs induced by viral infection inhibits the synthesis of viral proteins, exerting antiviral effects prior to the upregulation of host antiviral protein transcription. During infection, the double-stranded RNA (dsRNA) formed by the virus activates PKR, leading to eIF2α phosphorylation and subsequent promotion of SGs formation (McEwen et al., 2005; Yamasaki and Anderson, 2008). The RNA-binding protein GTPase-activating protein (SH3 domain)-binding protein 1 (G3BP1) binds to mRNA and aggregates, forming the core of SGs (Yamasaki and Anderson, 2008). Eukaryotic translation initiation factor 4G 1 (eIF4G1) is a key regulatory protein involved in translation initiation. G3BP1 can bind to and sequester eIF4G1, promoting stress-induced SGs formation and playing a role in mRNA transport and stability (Kedersha et al., 2016). Picornavirus 2A or L protein inhibits SGs formation by interfering with the eIF4GI-G3BP interaction (Yang et al., 2019). SVV infection induces the formation of stress granules (SGs) during the early stages of infection, but these SGs dissipate as the infection progresses to the late stages. Additionally, SVV infection transiently induces PKR and eIF2α phosphorylation-dependent SGs formation. SVV replication plays a crucial role in SGs formation. The cleavage of eIF4G1 by SVV. 3C^pro^ disrupts the eIF4GI-G3BP1 interaction, thus impairing SGs formation and contributing to enhanced viral replication (Wen et al., 2020).

### 3.4. The role of 3C protease in pyroptosis

The NLRP3 inflammasome, which is a large multiprotein complex that senses danger signals inside and outside the cell and initiates an inflammatory response, mainly consists of three components: NLRP3 (NOD-like receptor protein 3), ASC (apoptotic speck-like protein containing a caspase recruitment domain), and caspase-1. Upon the appearance of danger signals, such as pathogen infection, inflammatory stimulation or cell damage, the NLRP3 inflammasome can be activated, and once activated, the inflammasome can activate inflammatory cytokines such as IL-1β, IL-18 and gasdermin D (GSDMD) by stimulating caspase-1 activation. When activated, GSDMD forms pores and triggers pyroptosis, which is characterized by the rupture of the cell membrane and the release of intracellular contents. GSDMD belongs to the gasdermin family, which is a group of proteins with functions in cell membrane perforation and induction of inflammation. GSDMD has a self-inhibitory structure located between the N-and C-termini. When cleaved by proteases such as caspase-1, the N-terminus of GSDMD (GSDMD-N) is released and inserted into the cell membrane, forming pores. This process consequently results in the exchange of intracellular and extracellular substances, which ultimately leads to pyroptosis (Shi et al., 2015; Choudhury et al., 2022).

**Figure 2:**
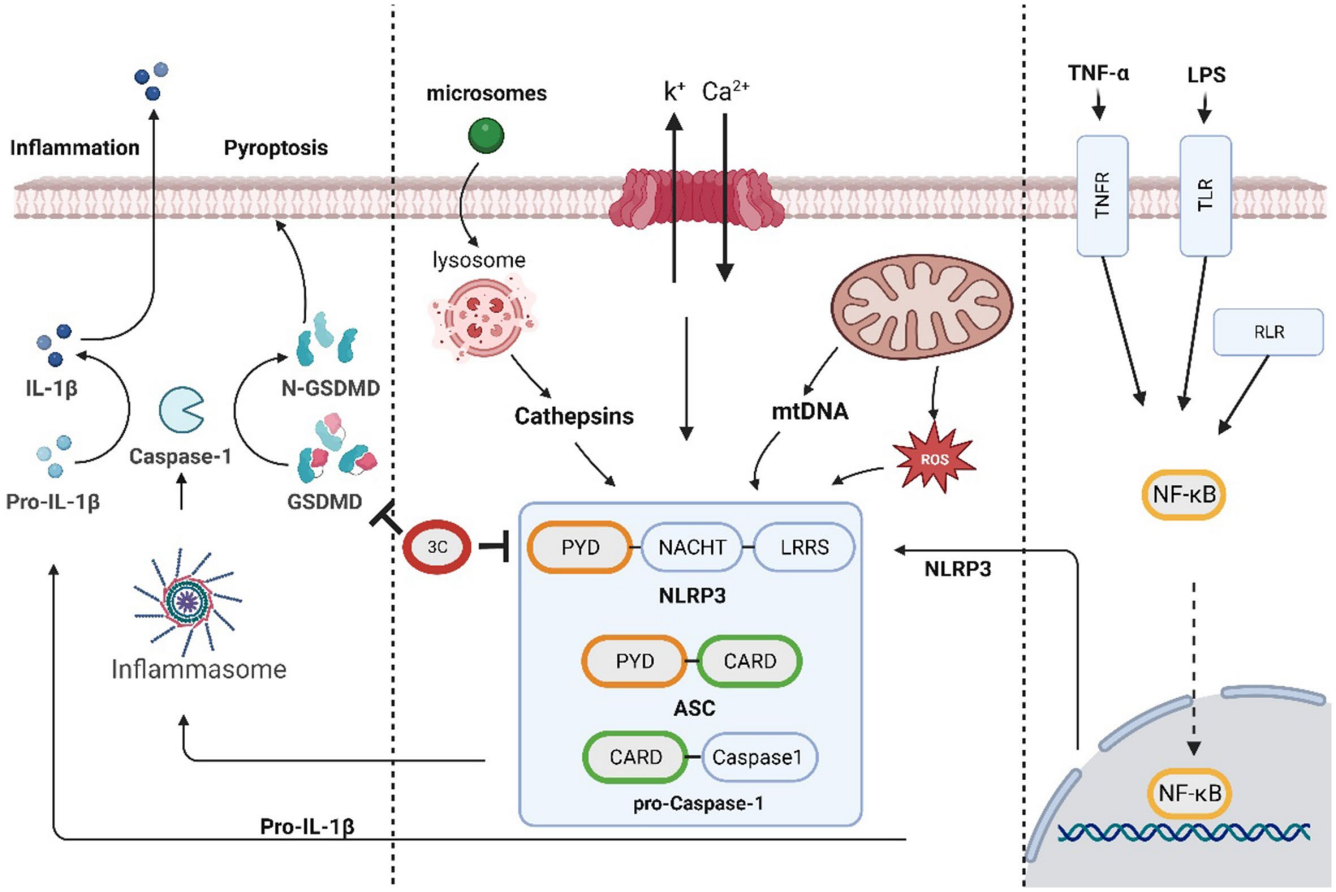
The SVV 3C protease is involved in the pyroptosis pathway and affects antiviral responses.

SVV 3C^pro^ cleaves NLRP3 at Q305, resulting in the inhibition of NLRP3 inflammasome formation. Concurrently, the protease directly cleaves pig GSDMD at Q193 and Q277, leading to release of the N-terminus of GSDMD. The cleavage of GSDMD 1–275 by caspase-1 triggers pyroptosis. Additionally, because GSDMD Q277 is proximal to the caspase-1-mediated cleavage site, GSDMD 1–277 may induce pyroptosis (Wen et al., 2021a). Research has shown that after infection with SVV markedly increases pig GSDMD cleavage and cell pyroptosis, confirming the inducible effect of SVV 3C^pro^ on GSDMD cleavage and pyroptosis. This finding suggests that SVV 3C^pro^ can independently trigger the inflammatory response of the NLRP3 inflammasome (Figure 2).

### 3.5. Role of 3C protease in apoptosis

Apoptosis is an essential programmed cell death mechanism involved in physiological and pathological processes, such as development, growth, immunity, and disease. Apoptosis is a widely recognized defence mechanism of the host and serves to ensure the death of cells that have been infected by viruses. There are two types of pathways that facilitate cell apoptosis, extrinsic and intrinsic, and these pathways are executed by the caspase family of cysteine proteases (Cohen, 1997; Elmore, 2007). The extrinsic pathway is initiated by external triggers and is regulated by membrane death receptors such as CD95 (Fas/APO-1) and tumour necrosis factor receptor 1 (TNFR1). When apoptosis is induced, caspase-8 is activated and triggers downstream effector molecules, including caspase-1, caspase-3, caspase-6, and caspase-7 (Muzio et al., 1996). The intrinsic pathway involves the activation of mitochondrial-related proteins. When host cells are subjected to factors such as infection and injury, the mitochondrial apoptosis pathway is stimulated, resulting in changes in mitochondrial membrane permeability and the release of various apoptotic factors, including cytochrome C (Galluzzi et al., 2012). This process leads to the activation of caspase-9 and downstream caspase-3, ultimately leading to cell apoptosis.

**Figure 3:**
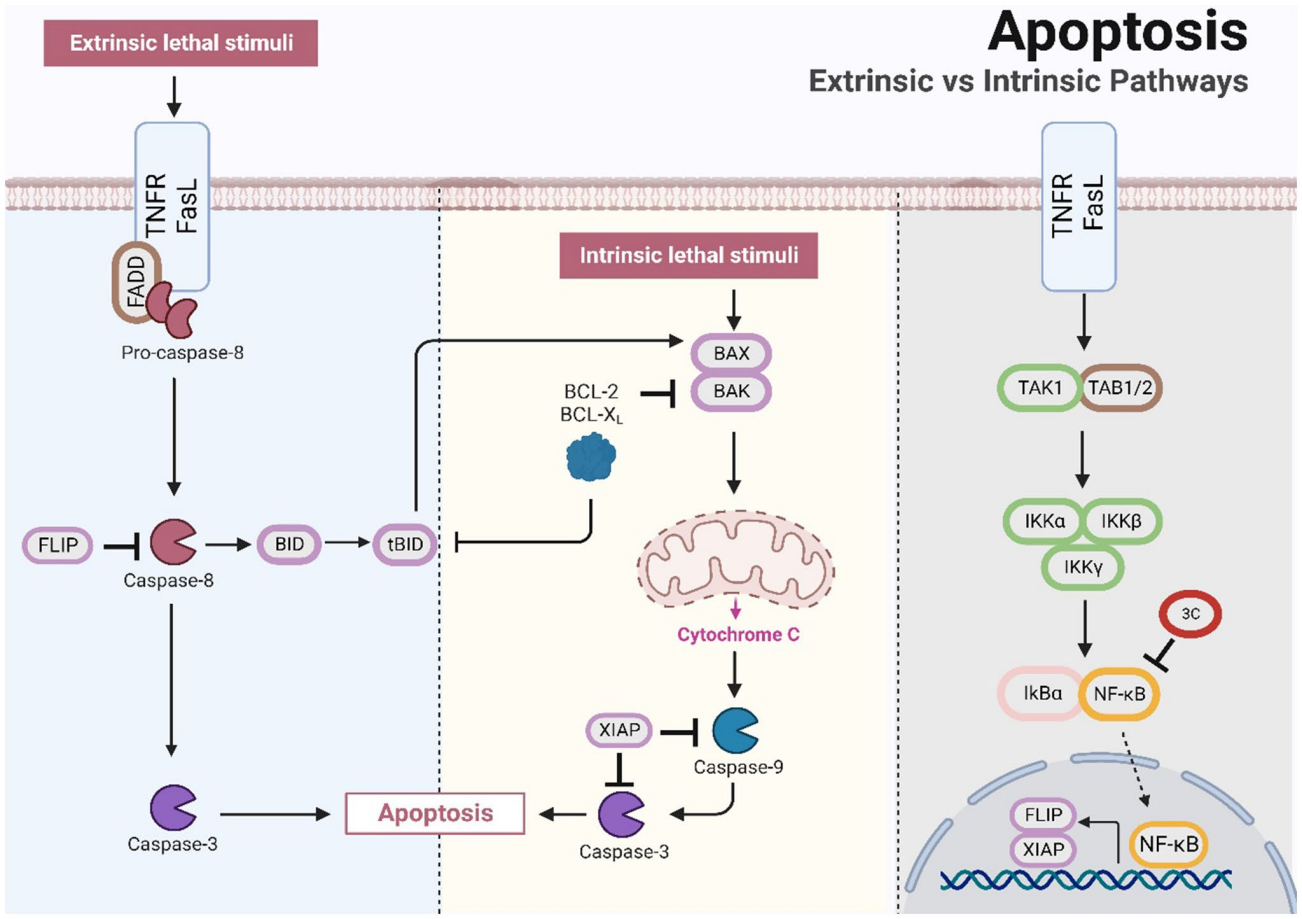
The SVV 3C protease is involved in the apoptotic pathway and affects antiviral responses.

At the early stages of SVA infection, the apoptotic pathway is not activated, which may allow the virus to complete its replication cycle before cell death/lysis and at the later stages of infection, the induction of cell apoptosis by SVV may function as a mechanism for facilitating the release and/or dissemination of the virus from infected cells (Kaminskyy and Zhivotovsky, 2010; Sun et al., 2016; Fernandes et al., 2019). The ability of 3C^pro^ to induce apoptosis in cells seems to remain consistent in small RNA viruses, including enterovirus 71 and poliovirus. Studies suggest that the SVV 3C^pro^ protein stimulates caspase-3, caspase-8, and caspase-9 and indicate that this protein invokes cell apoptosis through both mitochondrial and exogenous death receptor pathways (Liu et al., 2019). Although SVV 3C^pro^ induces cell apoptosis through its protease activity, it does not directly cleave PARP 1, which is a hallmark of cell apoptosis (Oliver et al., 1998; Liu et al., 2019). Emerging studies have shown that NF-κB likely plays a vital role in safeguarding host cells from small nuclear RNA virus-induced apoptosis (Oliver et al., 1998; Schwarz et al., 1998). A few studies have postulated that caspase 3-mediated cleavage of NF-κB encourages cell apoptosis (Kang et al., 2001; Kim et al., 2005), whereas SVV 3C^pro^ might impede innate immunity while promoting cell apoptosis by mediating the cleavage of caspase 3 through NF-κB (Fernandes et al., 2019; Figure 3). Notably, infection with human immunodeficiency virus (HIV) and African swine fever virus (ASFV) induces caspase-mediated NF-κB-p65 cleavage, thereby enhancing viral replication or inducing cell apoptosis after completion of the viral replication cycle (Vallée et al., 2001; Coiras et al., 2008). 3C^pro^ has a unique structural domain that binds to native phospholipid molecules, such as cardiolipin (CL) and phosphatidylinositol-4-phosphate (PI4P). CL can activate SVA 3C^pro^ activity in a homologous manner, leading to the cleavage of viral polyproteins and host proteins (such as NLRP3 and MAVS), disrupting host responses and ensuring viral replication. This binding serves as a positive regulatory mechanism for 3C^pro^ activity and promotes viral replication (Zhao et al., 2023). The replication of most positive-sense RNA viruses necessitates remodelling of cell membranes, converting them into virus replication organelles (ROs). The lipid microenvironment within ROs, which are rich in PI4P, is crucial for the replication of enterovirus RNA (Belov and van Kuppeveld, 2012). PV 3C^pro^ exhibits a wide and specific PIP-binding affinity for phospholipids, including PI4P. The binding of SVA 3C^pro^ to PI4P is hypothesized to induce membrane transformation and facilitate viral genome replication (Belov and van Kuppeveld, 2012; Zhao et al., 2023).

### 3.6. Role of 3C protease in autophagy

Autophagy is a cellular degradation process that breaks down proteins, organelles, and other substances into small molecules for cellular metabolism. During autophagy, the cell forms and releases autophagosomes containing degraded substances (Klionsky and Codogno, 2013). Modulating the mTOR pathway may affect the virus-induced autophagy response (Zhu et al., 2012). Autophagy enhancement can result from inhibiting AKT activation, whereas AKT activation can decrease autophagy (Hay and Sonenberg, 2004). AMP-activated protein kinase (AMPK) modulates autophagy by inhibiting the mTOR pathway. Phosphorylation of the mTOR binding partner Raptor, which is controlled by AMPK, is necessary to inhibit the mTOR pathway (Yang and Klionsky, 2010). The MAPK signalling pathway, which is essential in the signal transduction network of eukaryotes, plays a vital role in vital processes such as cell proliferation, differentiation, autophagy, apoptosis and the stress response (Gwinn et al., 2008).

A previous study revealed that SVV infection induces autophagy via the PKR-like ER protein kinase (PERK) and the activating transcription factor 6 (ATF6) signalling pathways associated with endoplasmic reticulum stress. Autophagy promotes SVV infection in pig cells, and the research results further demonstrate the involvement of the PERK and ATF6 pathways in autophagy induction. Decreasing the expression of PERK or ATF6 can inhibit SVV replication (Hou et al., 2019). However, in cultured human cells, SVV triggers an autophagic response that inhibits viral replication (Wen et al., 2021b). Research has demonstrated that VP1, VP3, and 3C^pro^ can cooperatively activate the AKT-AMPK-MAPK-mTOR signalling pathway during SVV infection in host cells, leading to the induction of cellular autophagy. The expression of 3C^pro^ upregulates the levels of p-ERK1/2 MAPK, p-p38 MAPK, and p-AKT and induces no significant change in the p-AMPK levels. The synergistic effect of p-ERK1/2 MAPK, p-p38 MAPK, and p-AKT causes a reduction in the p-MTOR levels. The transfection of cells with 3C^pro^ alone has no significant effect on the LC3-II level. However, transfection with SVV VP1 or 3D alone results in a significant alteration in the LC3-II level. VP1 expression promotes the phosphorylation of AKT and AMPK, leading to a reduction in the p-MTOR levels. Moreover, VP3 can activate the ERK1/2 MAPK-MTOR and p38 MAPK-MTOR pathways to facilitate autophagy. Therefore, the findings suggest that viral proteins can work synergistically to initiate autophagy, which in turn enhances viral replication (Song et al., 2022a). Currently, no studies have been investigated the impact of 3C^pro^ on the PERK and ATF6 UPR signalling pathways.

**Figure 4:**
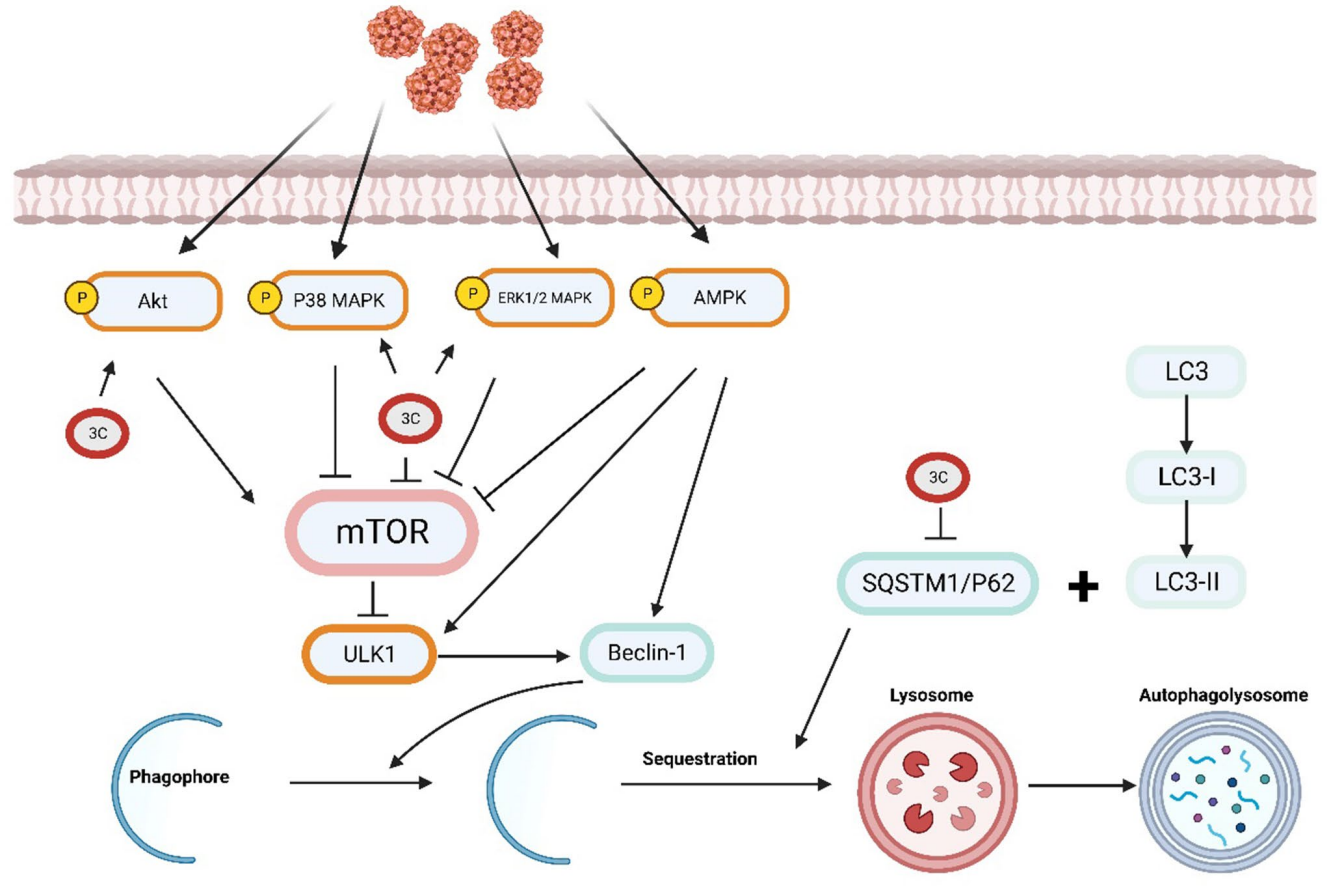
The SVV 3C protease is involved in the autophagy pathway and affects antiviral responses.

The receptor protein SQSTM1/p62 plays a significant role in the process of selective autophagy by recruiting ubiquitinated proteins or pathogens to autophagosomes for degradation (Bjørkøy et al., 2005; Lamark et al., 2017). Previous research reports have shown that SQSTM1 targets ubiquitin-dependent and ubiquitin-independent viral capsids for autophagic clearance, including FMDV, chikungunya virus (CHIKV) (Berryman et al., 2012; Judith et al., 2013). By targeting the SVV VP1 and VP3 proteins to autophagosomes, SQSTM1 suppresses viral replication. Research has revealed that SVV 3C^pro^ proactively cleaves SQSTM1/p62 at E355, Q392, and Q395, thereby producing an SQSTM1 cleavage product that cannot effectively induce selective autophagy or inhibit SVV replication (Wen et al., 2021b; Figure 4).

## 4. Conclusion

The 3C^pro^ of SVV is considered to play a significant role in SVV pathogenicity. This protease can affect host cell function through multiple mechanisms, inhibiting the immune response and promoting virus replication. Specifically, the protease can broadly inhibit the type I IFN pathway, enabling the virus to effectively evade host immune system attacks (Qian et al., 2017; Xue et al., 2018a,b; Wen et al., 2019). Additionally, SVV 3C^pro^ affects various processes, such as cell mRNA translation (Xue et al., 2020; Song et al., 2021, 2022b; Wen et al., 2022; Zhao et al., 2022), Pyroptosis (Wen et al., 2021a), apoptosis (Liu et al., 2019; Zhao et al., 2023), and autophagy (Wen et al., 2021b; Zhao et al., 2023), which may facilitate virus replication. Notably, this protease interacts with many crucial host proteins, cleaving them to exploit host cell resources while avoiding or mitigating immune responses (Table 1).

**Table 1:** Cleavage of cellular transcription factors by 3C^pro^.

Cellular target			Strategy			Reference	
Name	Impacted biological mechanism	Protein origin	Name	Site	Pathway	Title	Author
RIG-I	Viral sensing	Swine	Degradation	N.D	Caspase	Seneca Valley virus 2C and 3C inhibit type I interferon production by inducing the degradation of RIG-I	Wen et al. (2019)
TRIF	Viral sensing	Swine	Cleavage	Q159	N.D	Seneca Valley virus suppresses host type I interferon production by targeting adaptor proteins MAVS, TRIF, and TANK for cleavage	Qian et al. (2017)
MAVS	Signal transmitting	Swine	Cleavage	Q148	Caspase	Seneca Valley virus suppresses host type I interferon production by targeting adaptor proteins MAVS, TRIF, and TANK for cleavage	Qian et al. (2017)
TANK	Signal transmitting	Swine	Cleavage	E272/Q291	N.D	Seneca Valley virus suppresses host type I interferon production by targeting adaptor proteins MAVS, TRIF, and TANK for cleavage	Qian et al. (2017)
IRF3	Transcription	Swine	Degradation	N.D	N.D	Seneca Valley virus 3C^pro^ abrogates the IRF3- and IRF7-mediated innate immune response by degrading IRF3 and IRF7	Xue et al. (2018a)
IRF7	Transcription	Swine	Degradation	N.D	N.D	Seneca Valley virus 3C^pro^ abrogates the IRF3- and IRF7-mediated innate immune response by degrading IRF3 and IRF7	Xue et al. (2018a)
NF-κB	Transcription	Swine	Cleavage	444L-450R	Caspase-3	Senecavirus A 3C protease mediates host cell apoptosis late in infection	Fernandes et al. (2019)
eIF4G1	Translation	Swine	Cleavage	N.D	N.D	Seneca Valley virus 3C protease inhibits stress granule formation by disrupting eIF4GI-G3BP1 interaction	Wen et al. (2020)
G3BP1	Response amplification	Swine	Disrupting G3BP1-eIF4GI interaction	N.D	N.D	Seneca Valley virus 3C protease inhibits stress granule formation by disrupting eIF4GI-G3BP1 interaction	Wen et al. (2020)
PABPC1	Translation	Swine	Cleavage	Q437	N.D	Seneca Valley virus 3C^pro^ cleaves PABPC1 to promote viral replication	Xue et al. (2020)
DDX21	Viral sensing and signal transmitting	Swine	Degradation	N.D	Caspase	2B and 3C proteins of Senecavirus A antagonize the antiviral activity of DDX21 via the caspase-dependent degradation of DDX21	Zhao et al. (2022)
DHX30	Viral sensing and signal transmitting	Swine	Cleavage	Q220	N.D	Seneca Valley virus induces DHX30 cleavage to antagonize its antiviral effects	Wen et al. (2022)
hnRNP A1	Translation	Swine	Degradation	N.D	Proteasome	Seneca Valley virus 3C^pro^ degrades heterogeneous nuclear ribonucleoprotein A1 to facilitate viral replication	Song et al. (2021)
hnRNP K	Translation	Swine	Cleavage	Q364	N.D	Seneca Valley virus 3C^pro^ cleaves heterogeneous nuclear ribonucleoprotein K to facilitate viral replication	Song et al. (2022b)
hnRNP K	Translation	Swine	Degradation	N.D	Caspase	Seneca Valley virus 3C^pro^ cleaves heterogeneous nuclear ribonucleoprotein K to facilitate viral replication	Song et al. (2022b)
Nucleolin	Translation	Swine	Cleavage	Q545	N.D	Seneca Valley virus 3C^pro^ mediates cleavage and redistribution of nucleolin to facilitate viral replication	Song et al. (2022c)
NLRP3	Immune	Swine	Cleavage	Q305	Directly cleavage	Seneca Valley virus 3C protease induces pyroptosis by directly cleaving porcine gasdermin D	Wen et al. (2021a,b)
GSDMD	Immune	Swine	Cleavage	Q193/Q277	Directly cleavage	Seneca Valley virus 3C protease induces pyroptosis by directly cleaving porcine gasdermin D	Wen et al. (2021a,b)
SQSTM1	Target autophagosomes	Swine	Cleavage	E355/Q392/Q395	N.D	Selective autophagy receptor SQSTM1/p62 inhibits Seneca Valley virus replication by targeting viral VP1 and VP3	Wen et al. (2021b)
SQSTM1	Target autophagosomes	Human	Cleavage	N.D	N.D	Selective autophagy receptor SQSTM1/p62 inhibits Seneca Valley virus replication by targeting viral VP1 and VP3	Wen et al. (2021b)
SQSTM1	Target autophagosomes	Mouse	Cleavage	N.D	N.D	Selective autophagy receptor SQSTM1/p62 inhibits Seneca Valley virus replication by targeting viral VP1 and VP3	Wen et al. (2021b)

However, this review have several limitations. First, the referenced studies have heavily relied on *in vitro* cell experiments, and the results have not yet been validated in animal models. Second, investigating the existence of a synergistic effect between 3C^pro^ and other proteins has certain limitations. Furthermore, the described results from studies on the structural and functional changes of specific target proteins are not adequately comprehensive. Moreover, clarifying the differences in reactions among different hosts and cells is crucial. Additionally, due to limited research on the effects of SVV 3C protein on SGs, autophagy, and apoptosis, a comprehensive and in-depth understanding of its impact on these processes is still lacking.

It is crucial to examine how the virus employs 3C^pro^ to impair the host’s immune response. The related studies not only enhance our comprehension of the virus-and-host-cell interaction but also generate novel drug targets for antiviral agents. 3C^pro^ inhibitors restore the immune function of host cells and inhibit the replication of SVV. Several 3CL^pro^ inhibitors have been shown to inhibit coronavirus replication *in vitro* and enhance the survival rate of mice infected with Middle East respiratory syndrome coronavirus (MERS-CoV). Additionally, the capsid binder vapendavir and the newly developed 3C^pro^ inhibitor SG85 potently inhibit enterovirus 71 replication (Tijsma et al., 2014; Rathnayake et al., 2020). Similarly, understanding the impact of SVV 3C^pro^ on processes such as autophagy and apoptosis would expose additional details about virus-to-cell interactions., and this research outcome is vital for the development of new antiviral treatments. As an oncolytic virus, studying the ability of SVV to infect human cells and replicate, as well as the impact of 3C^pro^ on cellular immunity and programmed cell death, can provide a theoretical basis for tumour-related research in therapy (Reddy et al., 2007). The effect of SVV 3C^pro^ on the host is complex and interconnected and disrupts signal transduction, nuclear transport, transcription, translation, and protein stability. These effects are presumed to synergize with one another, but the underlying mechanisms require additional exploration.

## Author contributions

X-yZ and Y-yL jointly drafted the manuscript. H-xH, C-cZ, X-xL, B-pZ, and J-yL provided ideas that contributed to the conceptualization of this article. All authors contributed to the article and approved the submitted version.
